# Effects of Wheat and Oat-Based Whole Grain Foods on Serum Lipoprotein Size and Distribution in Overweight Middle Aged People: A Randomised Controlled Trial

**DOI:** 10.1371/journal.pone.0070436

**Published:** 2013-08-05

**Authors:** Paula Tighe, Garry Duthie, Julie Brittenden, Nicholas Vaughan, William Mutch, William G. Simpson, Susan Duthie, Graham W. Horgan, Frank Thies

**Affiliations:** 1 Division of Applied Medicine, University of Aberdeen, Aberdeen, United Kingdom; 2 Rowett Institute of Nutrition & Health, University of Aberdeen, Aberdeen, United Kingdom; 3 Clinical Biochemistry, National Health Service Grampian, Aberdeen, United Kingdom; 4 Biomathematics and Statistics Scotland, Rowett Institute of Nutrition & Health, Aberdeen, United Kingdom; Charité University Medicine Berlin, Germany

## Abstract

**Introduction:**

Epidemiological studies suggest three daily servings of whole-grain foods (WGF) might lower cardiovascular disease risk, at least partly by lowering serum lipid levels. We have assessed the effects of consuming three daily portions of wholegrain food (provided as wheat or a mixture of wheat and oats) on lipoprotein subclass size and concentration in a dietary randomised controlled trial involving middle aged healthy individuals.

**Methods:**

After a 4-week run-in period on a refined diet, volunteers were randomly allocated to a control (refined diet), wheat, or wheat + oats group for 12 weeks. Our servings were determined in order to significantly increase the intakes of non starch polysaccharides to the UK Dietary Reference Value of 18 g per day in the whole grain groups (18.5 g and 16.8 g per day in the wheat and wheat + oats groups respectively in comparison with 11.3 g per day in the control group). Outcome measures were serum lipoprotein subclasses' size and concentration. Habitual dietary intake was assessed prior and during the intervention. Of the 233 volunteers recruited, 24 withdrew and 3 were excluded.

**Results:**

At baseline, significant associations were found between lipoprotein size and subclasses' concentrations and some markers of cardiovascular risk such as insulin resistance, blood pressure and serum Inter cellular adhesion molecule 1 concentration. Furthermore, alcohol and vitamin C intake were positively associated with an anti-atherogenic lipoprotein profile, with regards to lipoprotein size and subclasses' distribution. However, none of the interventions with whole grain affected lipoprotein size and profile.

**Conclusion:**

Our results indicate that three portions of wholegrain foods, irrelevant of the type (wheat or oat-based) do not reduce cardiovascular risk by beneficially altering the size and distribution of lipoprotein subclasses.

**Trial Registration:**

www.Controlled-Trials.com ISRCTN 27657880.

## Introduction

High consumption of whole grain food (WGF) is associated with low risk of chronic disease such as coronary heart disease (CHD) [1], [2] and type 2 diabetes [3]–[6]. Despite inconsistent results, intervention studies using WGF or supplements of particular fibre components (psyllium, pectins, and gums) suggest that dietary whole-grain may protect against chronic diseases by altering serum lipid profiles [7], [8]. In the UK, WGF comprise mainly wheat, and to a lesser extent oats. The composition of micronutrients, fatty acids, and other phytochemicals differs between oats and wheat. Oat-based foods also contain high amounts of soluble fibers such as pectins, gums, and hemicelluloses, whereas wheat-based foods contain high amounts of insoluble fibres (mainly cellulose and insoluble hemicelluloses). This results in different glycaemic indices between diets containing oats and wheat, with oats higher than wheat. These differences in composition between both types of WGF seem to determine different serum lipaemic responses. Oats, and other foods containing high amount of soluble fibres, are effective in reducing plasma total and low density lipoprotein (LDL) cholesterol [8]–[10]. However, unlike wheat they do not alter triglyceride (TAG) levels [11]. We have recently shown that dietary interventions with either oats or wheat products have no significant beneficial effects on triglycerides (TAG) and total and LDL cholesterol [12]. However, a small scale study has suggested that oats effectively lower small, dense LDL cholesterol concentration and particle number without producing adverse changes in TAG or HDL-concentrations in middle-aged men [13]. Such an effect would partly explain why whole-grain could be beneficial against heart disease without changing total lipid profile. A comprehensive intervention trial was required to confirm these results. Most studies examining the effect of oats on cardiovascular disease (CVD) risk markers have been limited as they have used a wheat product as control [13]–[15] which does not allow the discrimination between the effects of refined or whole-grain wheat. In addition, the majority of studies carried out to date used β-glucan-enriched food or supplements [16]–[18]. Thus, a study that directly compares only whole grain wheat with refined wheat foods and oat-enriched diets is critical in order to elucidate the effects of these different grains on lipoprotein subclasses size and distribution.

In the UK, there is no specific dietary recommendation regarding whole-grain consumption [19]. Based on a meta-analysis of twelve population-based cohort studies [20], three servings per day of WGF could be sufficient to provide cardiovascular benefits. This study investigated the effects of a 12 week dietary supplementation with three servings per day of WGF (wheat provided as or a mixture of wheat and oats) on serum concentrations of lipoprotein subclasses as well as their size and distribution in free-living healthy middle-age volunteers.

## Subjects & Methods

The protocol for this trial and supporting CONSORT checklist are available as supporting information; see checklist S1 And protocol S1.

*Trial Registration:* isrctn.org identifier ISRCTN27657880.

### Participants

The study was conducted in concordance with CONSORT guidelines [21]. A single blind, randomised controlled dietary intervention study was carried out with men and women, aged 40–65 y with BMI between 18.5 and 35 kg/m^2^ recruited from the surrounding community of Aberdeen, Scotland. The study was approved by the North of Scotland Research Ethics Committee (04/S0801/066) and the volunteers gave written informed consent. Only subjects sedentary or moderately active (less than two aerobic sessions per week) were included. Individuals were also included if they presented signs of metabolic syndrome or moderate hypercholesterolemia. Individuals with CVD, diabetes or fasting blood glucose concentration >7.0 mmol/L, asthma, systolic blood pressure >160 mm or diastolic blood pressure >99 mm Hg, thyroid or eating disorders, with high habitual intake of WGF as well as people taking regular medication or supplements known to affect any dependant variable measured were excluded.

### Study design

Between June 2005 and September 2008, 233 participants from the surrounding area of Aberdeen were recruited to a 16-wk randomized, single blind, controlled parallel-designed trial involving three treatment groups (refined, wheat- and oat + wheat-based WGF). For the first 4 weeks, all volunteers consumed a refined diet to establish a baseline before allocation to the above treatment groups. The randomisation was delivered by the proven web/telephony randomisation system at the NIHR fully registered Clinical Trials Unit at the Centre for Healthcare Randomised Trials (CHaRT) at the University of Aberdeen. The algorithm used random permuted blocks stratified by age, gender and BMI. Compliance was determined by dietary assessment three times during the intervention (prior to run-in period, at baseline and during the intervention). The dietary interventions, practical and realistic for free-living individuals to achieve, were designed to compare a diet based on refined cereal products (refined cereals and white bread) with the substitution of 3 servings of refined cereals foods with 3 servings of whole wheat foods (70–80 g wholemeal bread +30–40 g whole grain cereals) or with the substitution of 3 servings of refined cereals foods with one servings of whole wheat foods (35–40 g wholemeal bread) and two of oats (60–80 g of whole grain rolled oats) provided as oatmeal and oat cakes (Patterson-Arran) containing 75% carbohydrate from oats and 9% olive oil as the only fat). Participants were provided with refined or wheat- or oat-based WGF widely available in the main UK food retailers. Our servings were determined in order to significantly increase the intakes of Non Starch Polysaccharides (NSP) to the UK Dietary Reference Value of 18 g per day (Department of Health, 1991). The participants were instructed not to alter their food intake, apart from the prescribed changes, and to maintain their usual level of physical activity and lifestyle. All measurements were performed four times, prior to the run-in period, at baseline, during and at completion of the intervention. In addition, participant's weight was monitored every two weeks during the dietary intervention and participants were asked to fill out a questionnaire about their health, level of exercise and medication. The samples obtained from the volunteers were anonymized and coded, and the people who assessed the outcomes (research assistants) were blinded after assignment to interventions.

### Dietary assessment

Dietary intakes were assessed prior to the onset of the study, during the 4 week run in and during the intervention period by means of a seven-day food diary. Subjects were given detailed advice, both written and oral, on how to complete the food diaries. The food diaries were then analysed for daily nutrient intakes using the dietary analysis program WISP (Version 3.0, Tinuviel Software, Warrington, UK).

### Blood pressure and anthropometric measurements

During each visit, volunteer's weight and height were measured for the determination of body mass index (BMI). Blood pressure was determined with an OMRON705CP sphygmomanometer with the subject seated, using the right arm and the appropriate size cuff. Blood pressure was measured at least one hour after the subjects' last meal and at least 30 min after smoking or consumption of caffeinated beverage. Subjects remained seated for 5 min prior to each measurement. Consecutive measurements (six on average) were carried out until the last three measurements showed less than 8% variation.

### Biochemistry

During each visit, 12-h fasted blood samples were taken from the antecubital fossa vein. Serum was prepared after centrifuging blood samples at 800 g at 4°C for 15 min and stored at −80°C until analysis. All samples were analysed in a single batch to reduce variability. VLDL, LDL and HDL subclasses concentrations and size in serum were determined by nuclear magnetic resonance (Liposcience Inc., Raleigh, USA). Data on intermediate density lipoprotein (22.7–27 nm) were not reported in this paper. The diameter range of the lipoprotein subclasses is shown in Table 1.

**Table 1:** Diameter range of lipoprotein subclasses.

LIPOPROTEIN	(nm)
**VLDL**	
Large VLDL	>60
Medium VLDL	35–60
Small VLDL	27–35
**LDL**	
Large LDL	21.2–23.0
Small LDL (total)	18.0–21.2
Medium Small LDL	19.8–21.2
Very Small LDL	18.0–19.8
**HDL**	
Large HDL	8.8–13.0
Medium HDL	8.2–8.8
Small HDL	7.3–8.2

### Statistical analysis

Statistical analyses were performed using the Statistical Package for the Social Sciences (version 17.0, SPSS Inc, Chicago, IL).

Data were analysed by using two-factor ANOVA of differences from baseline, with dietary group and gender as factors, and adjustment for age and BMI by including these as covariates. Where necessary because of non-Normally distributed errors, data values were log-transformed. P values for multiple comparisons between the three diets were adjusted by the Bonferroni method. Correlations between log-transformed data values were calculated using Pearson partial correlations corrected for BMI, age and energy intake. The sample size was originally estimated on the primary outcomes of total and LDL cholesterol concentrations. As cholesterol concentration variability between individuals has been found by other authors to be about 10%–20%, we assumed that baseline adjustment should reduce this to 5%–10%, indicating that 60 subjects per group would give sufficient experimental power (90%) to detect intervention effects of 5%–7%. Secondary outcomes were systemic markers of inflammation and lipoprotein subfraction size and concentrations.

## Results

**Figure 1:**
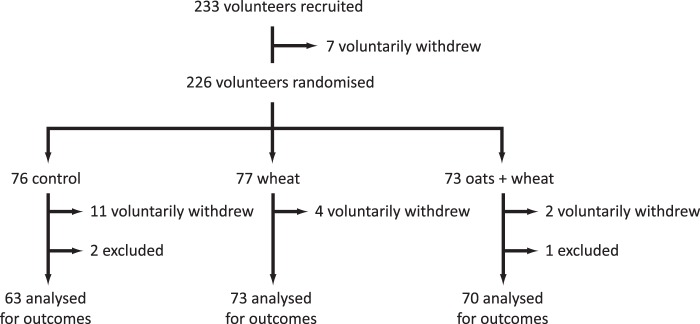
Trial profile.

A total of 233 volunteers were recruited. Of these 24 withdrew (9 for personal reasons, 3 for clinical reasons, 2 were unhappy with the group they were randomised to, 6 had digestive problems, 1 could not adhere to the protocol due to a desire to lose weight and 3 were lost to follow-up). Three volunteers did not meet inclusion criteria and were excluded from analysis. Therefore 206 participants completed the intervention (Figure 1).

The subject characteristics at baseline following 4 weeks run-in period were similar between the groups (Table 2). However, both systolic and diastolic blood pressures were significantly lower in the wheat group compared with the refined and wheat + oats groups. Serum lipid concentrations were similar between the groups, excepted for triglycerides which were significantly higher in the refined group compared with the wheat group, due to dissimilarities between male volunteers in these two groups. The weight of the volunteers remained unchanged during the course of the dietary intervention. For all groups, no significant differences in age, BMI, energy intake, systolic and diastolic blood pressures, and lipid levels were observed at baseline between those participants who completed the intervention and those who withdrew.

**Table 2:** Participant characteristics at baseline.^1^

	Refined			Wheat			Wheat + oats					
	*All*	*Men*	*Women*	*All*	*Men*	*Women*	*All*	*Men*	*Women*	P^2^	P^3^	P^4^
n	63	30	33	73	39	34	70	36	34			
Age (years)	51.8 (7.4)	50.9 (8.7)	53.2 (5.8)	51.6 (7.4)	50. 2 (7.4)	53.6 (6.9)	52.1 (7.4)	51.7 (7.4)	52.1 (7.4)	0.945	0.670	0.661
BMI (kg/m^2^)	28.0 (4.0)	28.5 (3.1)	27.6 (4.7)	28.0 (4.2)	28.5 (3.4)	27.5 (4.9)	27.0 (3.7)	27.1 (2.4)	26.9 (4.8)	0.221	0.085	0.815
Waist circum. (cm)	90.9 (12.1)	97.5 (8.4)	85.7 (12.2)	93.0 (12.2)	99.1 (9.7)	87.5 (11.9)	93.4 (12.1)	96.7 (8.2)	86.0 (13.1)	0.417	0.483	0.813
SBP 5 (mmHg)	131.2 (1.4)^a^	133.8 (2.8)^a^	128.9 (2.6)	125.9 (1.4)^b^	127.7 (2.0)^b^	123.7 (1.8)	131.7 (1.4)^a^	134.9 (1.9)^a^	128.3 (2.5)	0.019	0.041	0.242
DBP (mmHg)	79.1 (0.8)^a^	79.6 (1.5)	78.8 (1.4)^a^	75.7 (0.8)^b^	77.0 (1.4)	74.3 (1.2)^b^	78.4 (0.8)^a^	79.3 (1.1)^a^	77.6 (1.3)	0.026	0.315	0.053
Chol (mmol/L)	5.94 (1.11)	5.89 (1.04)	5.99 (1.12)	5.46 (1.18)	5.28 (1.01)	5.87 (1.30)	5.57 (1.03)	5.48 (0.95)	5.67 (1.12)	0.087	0.056	0.532
% change 5,6	−2.92 (7.69)^a^	−2.87 (9.31)	−2.97 (6.15)	2.23 (10.25)^b^	2.78 (11.42)	4.64 (8.95)	−0.40 (9.11)^ab^	0.33 (10.00)	−1.17 (8.15)	0.013	0.099	0.058
TAG^3^ (mmol/L)	1.49 (0.86)^a^	1.78 (1.03)^a^	1.23 (0.60)	1.27 (0.68)^ab^	1.44 (0.76)^ab^	1.08 (0.53)	1.12 (0.54)^b^	1.23 (0.49)^b^	1.00 (0.57)	0.012	0.024	0.222
% change^6^	0.13 (29.04)	3.32 (36.28)	−2.57 (21.32)	3.85 (32.35)	6.83 (37.22)	0.62 (26.22)	10.49 (31.07)	11.79 (35.21)	9.13 (26.45)	0.151	0.643	0.141
LDL chol (mmol/L)	3.66 (0.98)	3.68 (0.94)	3.65 (1.03)	3.45 (1.03)	3.30 (0.95)	3.62 (1.11)	3.45 (0.89)	3.47 (0.84)	3.43 (0.95)	0.365	0.250	0.652
% change^5,6^	−4.36 (10.48)^a^	−4.34 (11.01)^b^	−4.37 (10.18)^ab^	3.09 (13.78)	4.19 (14.70)	1.88 (12.81)	−1.89 (13.57)	−0.84 (16.20)	−3.00 (10.22)	0.007	0.059	0.058
HDL chol (mmol/L)	1.62 (0.48)	1.44 (0.42)	1.78 (0.48)	1.55 (0.40)	1.35 (0.30)	1.78 (0.38)	1.62 (0.40)	1.46 (0.33)	1.79 (0.41)	0.506	0.338	0.990
% change^6^	0.23 (11.42)	−1.10 (12.80)	1.37 (10.19)	2.92 (9.49)	3.00 (11.14)	2.84 (7.54)	3.58 (31.32)	6.16 (42.62)	0.85 (10.49)	0.615	0.575	0.668

1 Results are mean (SD), TAG = triglycerides.

2 Differences between groups (All) were assessed by using two-factor ANOVA. There was no significant diet-gender interaction.

3 Differences between groups for men were assessed by using one-factor ANOVA.

4 Differences between groups for women were assessed by using one-factor ANOVA.

5 Values in the same row with different superscript letters are significantly different (Bonferroni post hoc test).

6 % change from baseline after 12 weeks intervention.

Baseline energy and macronutrient intakes were similar for all groups and the results have been previously published [12]. The NSP daily intake reflected the average daily NSP intake in Scotland (2001/2 Expenditure and Food Survey [22]). There was no significant diet/gender interactions for the markers described in this paper and therefore only the overall results are presented without discriminating between genders.

Lipoprotein size and concentrations were measured by NMR and each lipoprotein fraction categorized into 3 to 5 different sub populations according to their size. Lipoprotein size was not significantly associated with any nutrient intake. However, some associations were found with lipoprotein subclasses' particle concentrations (Table 3). Alcohol intake was positively associated with total HDL and medium HDL particle concentration (R = 0.196, P<0.01 and R = 0.219, P<0.01 respectively), while an inverse relationship was found with the small HDL fraction (R = −0.189, P = 0.022). Total LDL particle concentration was negatively associated with vitamin C intake (R = 0.221, p = 0.007), mainly due to interactions with small LDL subclasses. Very low density lipoprotein (VLDL) concentrations were weakly associated with vitamin D intake, and this association was retained with medium VLDL subclass.

**Table 3:** Pearson partial correlation between lipoprotein subclasses concentration (nmol/L) and daily nutrient intake.

	Alcohol	Vitamin C	Vitamin D
	(g)	(mg)	(µg)
VLDL (Total)	−0.189^a^	0.062	−0.193^a^
Large VLDL	0.059	−0.077	0.149
Medium VLDL	−0.090	−0.103	−0.187^a^
Small VLDL	0.026	−0.048	−0.145
LDL (Total)	−0.042	−0.221^b^	−0.112
Large LDL	−0.11	−0.019	0.015
Small LDL (total)	−0.054	−0.236^b^	−0.069
Medium small LDL	−0.057	−0.219^b^	−0.067
Very small LDL	−0.052	−0.239^b^	−0.069
HDL (Total)	0.196^b^	−0.138	0.052
Large HDL	0.078	0.037	0.056
Medium HDL	0.219^b^	0.044	−0.097
Small HDL	−0.189^a^	0.144	0.125

Partial correlations at baseline (n = 206) were corrected for BMI, age and energy intake and calculated from log transformed values.

a P<0.05, ^b^P<0.01 (2-tailed).

The correlations at baseline between markers of inflammation and insulin resistance and the concentrations and size of the lipoprotein subclasses are shown in Table 4. Both LDL and HDL particle size were negatively correlated with systolic and diastolic blood pressures (P<0.01), independently of BMI and age. The concentrations of large VLDL and small LDL were positively associated with blood pressure (mainly diastolic). Lipoprotein size and subclasses' concentrations did not influence high sensitive C reactive protein (hsCRP), with the exception of LDL size and subclasses' concentrations which showed weak positive correlations with hsCRP (P<0.05, coefficient correlation <0.200). IL-6 showed no association with the particle size and subclasses' distribution apart from total HDL concentration (R = −0.221, P<0.01). Both LDL and HDL size were negatively correlated with ICAM-1 concentration (R = −0.175, P<0.05 and R = −0.215, p<0.01 respectively). The size of the VLDL lipoprotein fraction was not related to ICAM-1 concentration; however, all VLDL subclasses were positively associated with ICAM-1 concentration. LDL size was negatively associated with ICAM-1 concentration, small LDL sub fractions being also all negatively linked to this inflammatory marker. However, HDL showed no correlations with ICAM-1. Lipoprotein size seemed to affect insulin resistance as measured using the Homeostasis Model Assessment (HOMA). HOMA increased with VLDL particle size and decreased with LDL and HDL sizes. Lipoprotein subclasses concentrations were also significantly correlated with HOMA. VLDL and LDL concentrations, particularly the larger VLDL and smaller LDL particles, were positively associated with HOMA. Large HDL particle concentrations showed a negative correlation with insulin resistance while small HDL concentration was weakly but positively correlated with HOMA.

**Table 4:** Pearson partial correlation.

	SBP	DBP	hsCRP	ICAM-1	IL-6	HOMA
VLDL^1^	0.125	0.182^a^	−0.050	−0.054	−0.121	0.203^b^
LDL^1^	−0.223^b^	−0.247^b^	−0.192^a^	−0.175^a^	−0.104	−0.249^b^
HDL^1^	−0.236^b^	−0.227^b^	−0.115	−0.215^b^	0.050	−0.330^c^
VLDL (Total)^2^	0.144	0.166^a^	−0.026	0.295^c^	0.055	0.224^b^
LargeVLDL^2^	0.178^a^	0.218^b^	−0.023	0.183^a^	−0.098	0.246^b^
Medium VLDL^2^	0.066	0.060^a^	−.062	0.212^b^	−0.039	0.195^a^
Small VLDL^2^	0.100	0.096	−0.042	0.266^c^	−0.085	−0.183^a^
LDL (Total)^2^	0.143	0.208^b^	0.104	0.175^a^	0.079	0.230^b^
Large LDL^2^	−0.194^a^	−0.167^a^	0.154^a^	−0.090	−0.051	−0.180^a^
Small LDL (total)^2^	0.198^a^	0.228^b^	0.157^a^	0.169^a^	0.088	0.231^b^
Medium small LDL^2^	0.210^b^	0.241^b^	0.150	0.170^a^	0.071	0.241^b^
Very small LDL^2^	0.194^a^	0.223^b^	0.158^a^	0.167^a^	0.092	0.226^b^
HDL (Total)^2^	0.017	0.115	−0.161^a^	−0.092	−0.221^b^	−0.023
Large HDL^2^	−0.183^a^	−0.114	−0.084	−0.151	−0.048	−0.263^a^
Medium HDL^2^	0.187^a^	0.212^b^	−0.009	−0.112	−0.420	0.047
Small HDL^2^	0.091	0.086	−0.023	0.151	−0.146	0.196^a^

Partial correlations at baseline (n = 206) were corrected for BMI, age and energy intake and calculated from log transformed values.

a P<0.05, ^b^P<0.01, ^c^p<0.001 (2-tailed).

1 particle size (nM).

2 Particle concentration (nmol/L).

### Effect of intervention on lipoprotein subclasses concentrations and size

VLDL mean size was significantly higher in the refined group compared with both whole grain groups, at baseline and after 12 week intervention (Table 5). This reflect a higher concentration of large VLDL and relates to the significantly higher serum triglyceride concentrations observed in the refined group as VLDL size is mainly determined by triglyceride content. Furthermore, men from the refined group had a significantly higher triglyceride concentration compared with the men from the wheat + oats groups. These results were associated with concomitant differences between the same groups in small LDL and large VLDL concentrations (p = 0.048 and p = 0.013 respectively, results not shown). This confirms previous findings suggesting that serum triglyceride levels >1.5 mmol/l are a predictor of large VLDL and small LDL.

**Table 5:** Lipoprotein size (nm) in response to 12 weeks intervention with refined, wheat- or wheat + oats – based diets^1^.

		Refined	Wheat	Wheat + oats	P^2^	P^3^
		(n = 63)	(n = 73)	(n = 70)		
VLDL	Wk4^4^	50.81 (1.35)^a^	47.95 (1.19)^a,b^	47.52 (0.85)^b^	0.032	
	Wk16	51.02 (1.27)	48.36 (1.13)	47.39 (0.83)		
	Diff.^5^	0.21 (1.27)	0.42 (1.29)	−0.16 (0.81)		0.933
LDL	Wk4	21.16 (0.12)	21.27 (0.09)	21.27 (0.09)	0.273	
	Wk16	21.20 (0.11)	21.29 (0.11)	21.39 (0.08)		
	Diff.	0.04 (0.07)	0.02 (0.07)	−0.12 (0.06)		0.782
HDL	Wk4	9.08 (0.07)	9.14 (0.07)	9.22 (0.05)	0.298	
	Wk16	9.10 (0.08)	9.13 (0.06)	9.18 (0.06)		
	Diff.	0.02 (0.03)	−0.01 (0.02)	−0.04 (0.02)		0.562

1 Values are mean (SEM).

2 Differences at baseline between groups were assessed by using two-factor ANOVA on log-transformed values.

3 Differences in size change from baseline between the dietary intervention groups were assessed by using two factor ANOVA on log transformed values.

4 Values in the same row with different superscript letters are significantly different (Bonferroni post hoc test).

5 Represents differences calculated as mean concentration at week 16 – mean concentration at week 4.

However, none of the dietary interventions affected significantly the distribution of lipoprotein subclasses. Size (table 5) and particle concentrations of VLDL, HDL and LDL subclasses (Tables 6, 7 and 8 respectively) were not significantly affected by the intervention.

**Table 6:** VLDL subclasses particle concentrations (nmol/L) in response to 12 weeks intervention with refined, wheat- or wheat + oats – based diets^1^.

		Refined	Wheat	Wheat + oats	*P* 2	*P* 3
		(n = 63)	(n = 73)	(n = 70)		
VLDL (Total)	Wk4	66.57 (5.11)	57.89 (3.86)	53.98 (3.84)	0.217	
	Wk16	61.83 (4.95)	56.85 (3.72)	57.90 (4.05)		
	Diff.^4^	−4.77 (2.21)	−1.03 (2.57)	3.92 (2.42)		0.158
Large VLDL	Wk4	3.78 (0.71)	2.53 (0.40)	2.08 (0.34)	0.121	
	Wk16	3.75 (0.66)	2.48 (0.42)	2.39 (0.41)		
	Diff.	−0.03 (0.38)	0.06 (0.40)	0.31 (0.27)		0.864
Medium VLDL	Wk4	24.52 (2.59)	20.93 (1.84)	16.88 (1.79)	0.057	
	Wk16	24.32 (2.99)	18.48 (1.82)	20.67 (2.3)		
	Diff.	−0.20 (2.33)	−2.45 (1.73)	3.79 (1.77)		0.053
Small VLDL	Wk4	38.25 (3.2)	34.42 (2.39)	35.01 (2.22)	0.513	
	Wk16	33.74 (2.6)	35.88 (2.27)	34.84 (2.40)		
	Diff.	−4.51 (2.24)	1.46 (1.76)	−0.18 (1.69)		0.152

1 Values are presented as mean (SEM) or (SED) for differences between week 16 and week 4.

2 Differences at baseline between groups were assessed by using two-factor ANOVA on log-transformed values.

3 Differences in concentration change from baseline between the dietary intervention groups were assessed by using two-factor ANOVA.

4 Represents differences calculated as mean concentration at week 16 – mean concentration at week 4.

**Table 7:** LDL subclasses particle concentration (nmol/L) in response to 12 weeks intervention with refined, wheat- or wheat + oats – based diets^1^.

		Refined	Wheat	Wheat + oats	*P* 2	*P* 3
		(*n* = 63)	(*n* = 73)	(*n* = 70)		
LDL (Total)	Wk4	1326 (58)	1188 (48)	1164 (47)	0.075	
	Wk16	1277 (53)	1211 (46)	1175 (47)		
	Diff.^4^	−49 (30)	23 (28)	10 (23)		0.276
Large LDL	Wk4	508 (30)	485 (26)	503 (21)	0.574	
	Wk16	505 (33)	503 (32)	466 (22)		
	Diff.	−3 (24)	18 (19)	−37 (14)		0.111
Small LDL (Total)	Wk4	775 (67)	659 (50)	614 (45)	0.431	
	Wk16	731 (63)	668 (55)	663 (50)		
	Diff.	−44 (41)	9 (36)	49 (28)		0.261
Medium Small LDL	Wk4	157 (13)	134 (10)	127 (9)	0.327	
	Wk16	152 (13)	139 (12)	135 (10)		
	Diff.	−5 (9)	5 (8)	8 (6)		0.583
Very Small LDL	Wk4	618 (54)	524 (40)	487 (36)	0.121	
	Wk16	579 (51)	529 (44)	528 (40)		
	Diff.	−39 (33)	5 (28)	40 (22)		0.206

1 Values are presented as mean (SEM) or (SED) for differences between week 16 and week 4.

2 Differences at baseline between groups were assessed by using two-factor ANOVA on log-transformed values.

3 Differences in size change from baseline between the dietary intervention groups were assessed by using two-factor ANOVA.

4 Represents differences calculated as mean concentration at week 16 – mean concentration at week 4.

**Table 8:** HDL subclasses particle concentration (nmol/L) in response to 12 weeks intervention with refined, wheat- or wheat + oats – based diets^1^.

		Refined *(n* = 63)	Wheat (*n* = 73)	Wheat + oats (*n* = 70)	*P* 2	*P* 3
HDL (Total)	Wk4	32.1 (0.8)	30.1 (0.8)	31.2 (0.6)	0.155	
	Wk16	31.9 (0.7)	31.2 (0.5)	31.9 (0.5)		
	Diff.^4^	−0.1 (0.4)	1.0 (0.4)	0.6 (0.4)		0.155
Large HDL	Wk4	7.9 (0.6)	8.1 (0.5)	8.4 (0.4)	0.216	
	Wk16	8.1 (0.6)	8.2 (0.5)	8.4 (0.5)		
	Diff.	0.2 (0.2)	0.1 (0.2)	−0.1 (0.2)		0.715
Medium HDL	Wk4	3.8(0.5)	3.8 (0.5)	5.0 (0.5)	0.130	
	Wk16	4.2 (0.5)	4.3 (0.5)	4.4 (0.5)		
	Diff.	0.4 (0.4)	0.5 (0.4)	−0.6 (0.4)		0.110
Small HDL	Wk4	20.4 (0.8)	18.2 (0.7)	17.8 (0.7)	0.282	
	Wk16	19.6 (0.9)	18.7 (0.6)	19.1 (0.8)		
	Diff.	−0.8 (0.6)	0.5 (0.6)	1.3 (0.5)		0.053

1 Values are presented as mean (SEM) or (SED) for differences between week 16 and week 4.

2 Differences at baseline between groups were assessed by using two-factor ANOVA on log-transformed values.

3 Differences in concentration change from baseline between the dietary intervention groups were assessed by using two-factor ANOVA on log-transformed values.

4 Represents differences calculated as mean concentration at week 16 – mean concentration at week 4.

## Discussion

This is the first time to our knowledge that the differential effects of dietary oats and wheat on lipoprotein size and distribution have been comprehensively studied. The dietary interventions were practical and realistic for free-living individuals to achieve.

The associations found between lipoproteins sizes and subclasses' concentrations and various markers of CVD risk are interesting. Our results suggest a beneficial association between LDL and HDL sizes and blood pressure, in support with the findings from the Framingham Heart study which found similar correlations in volunteers with metabolic syndrome [23]. The relationship between systemic inflammatory markers and lipoprotein size is not well documented; a high concentration of small dense LDL is often concomitant with elevated inflammatory marker concentration in patients with metabolic syndrome and type II diabetes [24]–[26] However, lipoprotein size and subclasses' concentration were not associated with the inflammatory markers hsCRP and IL-6 measured in this study, as previously shown in HIV-infected patients [27]. The inverse correlation found between LDL and HDL sizes and ICAM-1 concentration underlines the potential benefit of having larger, buoyant LDL and HDL particles compared with small and dense one. Indeed a decreased HDL particle size is associated with an adverse cardiometabolic risk profile in healthy middle-aged men and women [28]. While the size of the VLDL particle was not linked to ICAM-1 concentrations, all VLDL subclasses were positively associated with ICAM-1 concentration, underlining the potential proinflammatory and proatherogenic implications of hypertriglyceridemia.

Small LDL concentrations were inversely associated with ICAM-1 concentration, further suggesting the negative impact of that class of particle in the atherogenic processes. Similar findings were previously observed in boys [29]. However, HDL particle concentration showed no correlations with ICAM-1 in contrast to that observed in HIV-infected patients [27].

The strongest interactions were found with HOMA, a marker of insulin resistance. Both size and lipoproteins particle subclasses concentrations showed significant correlations, both detrimental (VLDL particle size) and beneficial (LDL and HDL sizes) with insulin resistance, as previously shown in subjects with diabetes [30] and in children [31]–[33]. Lipoprotein subclasses concentrations were also significantly correlated with HOMA, the larger VLDL and smaller LDL and HDL particles being positively associated with increased insulin resistance while the opposite relationship was observed with large HDL particle concentrations. Interestingly, a previous study [34] showed that adiponectin, which is positively linked with insulin sensitivity [35], was inversely associated with atherogenic lipoprotein profile, even after adjustment for obesity and insulin resistance as measured using HOMA. However, this marker was not measured in our study.

Lipoprotein size was not significantly associated with any macro- and micronutrient intake, as previously shown in non-diabetic subjects [36], [37] However, alcohol intake was positively associated with HDL particle concentration, due to a significant correlation with larger HDL particle. A similar association has been recently described [38] in an elderly population, suggesting that moderate consumption of alcohol could reduce CVD risk by favourably changing the lipoprotein profile. Interestingly, vitamin C may also be beneficial against CVD risk as its intake was negatively associated with total LDL particle concentration, particularly with small LDL subclasses. A meta-analysis of 13 randomized controlled trials showed that daily supplementation with at least 500 mg/d of vitamin C, for a minimum of 4 weeks, can significantly decrease serum LDL cholesterol and triglyceride concentrations in hypercholesterolemic patients [39]. However this is, to our knowledge, the first time that a link between vitamin C intake and LDL subclasses concentrations has been reported, and such association would be worthy of further investigation. Possible mechanisms include the inhibition of LDL oxidation, which could preserve the ability of LDL to be recognised and removed from the circulation by LDL receptors [39]. Vitamin C could also promote LDL receptor activity [39] or modulate cholesterol ester transfer protein activity. The positive association between VLDL concentration and vitamin D intake could be explained by the fact that vitamin D binding protein and 25(OH)-vitamin D(3) are present in VLDL [40]. More VLDL would mean more vitamin D binding protein circulating within these lipoprotein particles and therefore would increase the amount of vitamin D associated.

This study primarily aimed to determine the effects of an intervention with wheat and oats on lipoprotein size and subclasses' concentrations. Indeed oats and soluble fibres such as β-glucan seem effective in reducing serum cholesterol concentration, as demonstrated by the results of many intervention studies using β glucan-enriched food or supplements, especially in hypercholesterolemic subjects, have been carried out over the last 10 years [16]–[18], [41]. An intake of β-glucan of 3 g/day (equivalent to around 60 g oatmeal) appears to be the minimum amount required to achieve a clinically-relevant decrease in serum cholesterol concentration [42]. While we were providing to our volunteers a similar amount of oats (60 to 80 g oatmeal/day), our results showed no effect of oat + wheat on blood lipid concentrations [12]. Such a lack of effect may be ascribed in part to the health status of our subjects who were not severely hypercholesterolemic. The WHOLEheart trial [43], another comprehensive 16-wk intervention trial with whole grains, also showed no beneficial effects on blood lipids. However, our previous results showed a significant reduction in total cholesterol and LDL cholesterol concentrations after a 12 week intervention in the refined group compared with the wheat group, independent of changes in either body weight or any dietary factors known to affect serum cholesterol concentrations [12]. However, the changes observed in cholesterol concentration in the wheat group were not supported by the results obtained on lipoprotein subclasses. Lipoprotein particle number and size, particularly for LDL, are strong predictors of CVD [44]–[46] and provide an independent measure of atherogenicity which may be superior to total cholesterol determination. However, none of the dietary interventions significantly altered the size and concentrations of lipoprotein particles. This is in contrast to previous findings [13] in overweight men, where lipoprotein subclasses patterns were modified favourably following the consumption of 14 g dietary fibre/day (as oats) for 12 weeks. The authors reported that oat consumption induced a 17% decrease in small, dense LDL cholesterol concentrations and particle number without altering serum triglyceride and HDL-cholesterol concentrations. Furthermore, consumption of wheat cereals led to a non significant reduction in LDL size and a significant increase (60%) in small LDL concentration. However, we found that LDL subclasses size and distribution were unaffected by any of the dietary interventions, even when considering men and women separately. Our trial included a refined group as control, unlike the study described above. Furthermore, the type as well as the total amount of cereals ingested by the volunteers also differed, which might account for these contradictory results.

The lipoprotein results reported here were not the primary outcome of the trial in which they were observed. As such, some caution is needed in evaluating them. When many outcomes are examined, the risk of a type I error (false positive) is increased. As they were not independent, a Bonferroni adjustment would have increased the type II error risk excessively [47]. However, we have observed several significant associations, many with p<0.01, and it is therefore unlikely that all could arise from type I errors. Although the power calculation was done for the primary outcome and not for the secondary results we have considered, this does not impact on the positive findings.

Our results indicate that three portions of WGF, irrespective of the type (wheat or oat-based) do not reduce cardiovascular risk by beneficially altering the lipoprotein profile.

## Supporting Information

Checklist S1 **CONSORT Checklist.**
(DOCX)

Protocol S1 **Trial Protocol.**
(DOC)
