# Supplementary material for: Effects of Wheat and Oat-Based Whole Grain Foods on Serum Lipoprotein Size and Distribution in Overweight Middle Aged People: A Randomised Controlled Trial
Source: PLoS One. 2013 Aug 5;8(8):e70436. doi: 10.1371/journal.pone.0070436 (PMC3734251; doi:10.1371/journal.pone.0070436)
Supplement: Protocol S1 — Trial Protocol. (DOC) [file pone.0070436.s002.doc]

**Effects of whole grain foods on markers of cardiovascular risk**

**REC reference number: 04/S0801/66**

| The aims of this project are (1) to test the hypothesis that three servings of whole grain foods per day may have a cardio-protective role and (2) to characterise the effects of this intervention on cardiovascular risk factors. The proposed study will also compare the effects of wheat-based whole grain foods with a mixture of wheat- and oat-based whole grain foods.  As Scotland has one of highest rates of mortality from cardiovascular disease (CVD) in Europe this project presents an opportunity to determine the effects of dietary intake of various whole grain foods on CVD risk in a population known to have high levels of markers for cardiovascular risk compared with elsewhere in the UK.  By choosing overweight people aged 40-65 years we aim to target people in whom the atherosclerotic processes and other pathologies related to CVD start to be more prominent, but are still reversible compared with older populations, where the disease is fully established and resistant to treatment by dietary intervention.  This project is designed to precisely meet the current FSA stated requirements for randomised controlled dietary intervention trials to investigate the effects of whole grain foods on CVD risk, as determined by measures of insulin sensitivity, lipidaemia, markers for inflammation and vascular function.  **Background:**  CVD is the main cause of mortality in Western countries, and particularly in Scotland where 338 men and 129 women per 100,000 die every year from CVD (1). The disease has a multi-factorial basis of which dietary factors represent a key component. There is strong epidemiological evidence that high consumption of whole-grain food is associated with low risk of chronic disease such as coronary heart disease (CHD) (2,3). A meta-analysis of twelve population-based cohort studies (4) recently concluded that individuals with the highest intake of whole grains have a lower risk of developing CHD compared with individuals with the lowest intake of whole-grain food. Moreover, whole-grain food intake had a stronger association with protection from CHD than intake of cereals, fibre, vegetables or fruits. The authors concluded that three servings of whole-grains per day might have an important cardio-protective effect.  A recent comprehensive observational study also observed an inverse association between cereal fibre intake and peripheral arterial disease risk in men (5). Several risks factors for CVD have been identified, and some are reversible and can be altered by dietary intervention. High plasma cholesterol, hypertension, high fasting triglycerides (TAG), low High Density Lipoprotein (HDL) cholesterol, diabetes and obesity are well recognized. Other implicated factors are low antioxidant status, hyperhomocysteinemia and evidence of inflammatory state (6). Certain markers such as cholesterol (LDL, HDL), C reactive protein (CRP) and blood pressure are particularly valuable predictors of CVD risk. Several randomized controlled trials showed that increase intake of whole-grain food were associated with a reduced risk of progression of impaired glucose tolerance to type 2 diabetes (7,8,9,10). Clinical trials confirmed these data showing that whole-grain intake increases insulin sensitivity (11). Recent studies have also found a negative association between high consumption of whole-grain food with prevalence of diabetes (9,10). Clinical trials confirmed these data showing that whole-grain intake increases insulin sensitivity (11). Whole-grain food also seems effective in reducing hypertension (12). Hyperhomocysteinemia has been associated with increase risk of CHD and cerebral vascular disease (13). Whole-grains contain folate and pyridoxine both of which are associated with reductions in plasma homocysteine levels (14). It has been suggested that whole-grain foods might also have protective effects on blood vessels, independently of their lipid lowering effect, through the phytochemicals (phytooestrogens) they contain (15).  Clearly, whole-grain foods have the potential to alter the main risk factors for CVD, but also vascular reactivity. Many dietary intervention studies using different types of whole-grain foods or using supplements of particular components of fibres (psyllium, pectins, and gums) have been carried out in animal models, but fewer in humans. However, the recommendation of three servings per day as protective for CVD risk is based mainly on information collected from meta-analyses of epidemiological studies but this has as yet not been properly tested with an intervention trial.  Current UK dietary advice regarding the consumption of whole grain foods is contained within more general advice to increase the consumption of complex carbohydrates (16). The FSA currently advise the public to choose wholemeal, brown or high fibre varieties of bread and cereals whenever possible but there is no advice regarding the quantity of this group of foods in the same way as there is for fruit and vegetables. In Scotland, dietary targets have been set to increase bread consumption by 45% to 153g per day by 2005 and to increase the consumption of breakfast cereal (though type is not specified) (16). Dietary trends in the National Food Survey data for Scotland (1994-2000) indicate that although consumption of both bread and breakfast cereals may have increased slightly, consumption of whole grain varieties remains very low. A recent analysis of wholegrain food consumption in the 1986/7 National Diet and Nutrition Survey of British Adults showed that the median consumption of whole grains was just 3 servings per week, while a similar analysis of the 1994/5 survey of people aged 65 and over showed the intake increased to 5 servings per week (17). With low intakes of whole grain foods wide-spread and emerging epidemiological evidence indicating that increasing the intake of wholegrain foods to three servings per day may be protective against CVD this project is timely and will provide the evidence base for new dietary advice to the UK public.  Whole-grain foods are rich sources of a variety of nutrients including carbohydrates, protein and essential fatty acids as well as a variety of micronutrients (vitamins and trace elements), antioxidants, phytochemicals, resistant starch and fiber (18). Thus, specific nutrients or particular combinations of several nutrients found in whole-grain foods could be responsible for the observed protective effects against CVD. They may protect against such chronic diseases by altering serum cholesterol profiles, exerting antioxidant properties and anti-thrombotic action, and through their beneficial effects on vascular reactivity and insulin sensitivity (19).  In the UK, whole-grain foods comprise mainly wheat, and to a lesser extent oats. The composition of micronutrients, fatty acids, and other phytochemicals differs between oats and wheat. Oat-based food also contains high amounts of soluble fibers such as pectins, gums, and hemicelluloses whereas wheat-based food contains high amounts of insoluble fibers (mainly cellulose and insoluble hemicelluloses). This results in different glycaemic indices between diets containing oats and wheat, with oats higher than wheat. These differences in composition between both types of whole-grain food seem to determine different serum lipaemic responses. Oats, and other foods containing high amount of soluble fibers are effective in reducing plasma total and LDL cholesterol (20,21,22), HDL cholesterol concentration also being slightly reduced. However, they do not affect triglyceride levels unlike wheats, which seem to have potent triglyceride-lowering effect (19). Some studies have reported no significant effect of oats on total cholesterol levels. However, oats were shown to effectively lower concentrations of small, dense LDL cholesterol (the most atherogenic one) and particle number without producing adverse changes in TAG or HDL-concentrations (23). These alterations may explain the cardioprotective effects of oats. The development of atherosclerotic plaques, a key event involved in the development of CVD and atherosclerosis, is now well recognized as an inflammatory disease (24). Inflammation plays an important role in the pathogenesis of plaque formation, as indicated by the infiltration and presence of activated T-lymphocytes, macrophages and mast cells in the atherosclerotic lesion, and by increased serum levels of prostanoids and C-reactive protein. Phytochemicals and certain minerals present in wholegrain food appear to exhibit anti-inflammatory, anti-aggregatory and anti-thrombotic properties (6,16,25,26) that would reduce plaque formation.  In summary, although there is a recommendation to consume three servings per day of whole-grain food in order to reduce the risk of CVD, the validity of such a recommendation remains to be substantiated scientifically. Furthermore, the quality of whole-grain seems to be important with regard to the effect on some CVD markers such as serum cholesterol and TAG levels. Therefore, the proposed study will investigate the effects of dietary supplementation with three servings per day of whole-grain food provided as a mixture of wheat and oats or only wheat on markers for cardiovascular risk, like inflammation and endothelial activation in middle-age volunteers. The primary end-points will be serum total and LDL cholesterol concentrations, as well as insulin sensitivity. The secondary end-points will be the measurement of vascular function and inflammation markers (vascular tonicity and function by pulse-wave velocity, von Willebrand factor and arginine and methylated metabolites, ICAM-1, IL-6, hsCRP and fibrinogen)**.** The findings of this study will provide a sound scientific basis for the justification of increasing whole-grain intake in nutritional policies to combat cardiovascular diseases. |
| --- |

| **PART B – DESCRIPTION OF SCIENTIFIC / TECHNOLOGICAL OBJECTIVES AND WORKPLAN** |
| --- |

**If the proposal is successful, information detailed here will form the Scope of Work section of the research contract.**

| **B1. Objectives and Expected Achievements** | |
| --- | --- |
|  | |
| **Objective No.** | **Objective Description** |
| 01 | To recruit and train new staff and recruit volunteers aged 40-65 and assess their characteristics |
| 02 | To assess the individual subject dietary intake and its relation to levels of cardiovascular risk factors, before, during and after dietary intervention. |
| 03 | To determine the effects of the diets on vascular function, as measured in vivo by pulse-wave velocity. |
| 04 | To determine the effects of the diets on serum cholesterol (total, HDL and LDL), triglycerides, apoA1and apoB levels |
| 05 | To determine the effects of the diets on lipoprotein subclasses size and number. |
| 06 | To determine the effects of the diets on insulin sensitivity, as measured indirectly using mathematical combinations of fasting insulin, glucose with or without non esterified fatty acid measures (logHOMA-IR and revised-QUICKI respectively). |
| 06 | To determine the effects of the diets on plasma **ghrelin, arginine and methylated metabolites,** homocysteine and folate levels. |
| 07 | To determine the effects of the diets on marker of inflammation. C reactive protein, IL-6 , ICAM-1**, von Willebrand factor and fibrinogen** will be measured. |
| 08 | **To determine the effects of the diets on bone markers (N-terminal propeptide of type 1 collagen (P1NP), serum beta C-terminal telopeptide (CTX) and osteaocalcin and enterolactone plasma concentration** |

| **B2. Approaches and Research Plan** |
| --- |
|  |
| A summary of the study design and experimental procedure is given in annexes 1 and 2.  **Study design:**  It is a single blind, randomised controlled longitudinal study involving three treatment groups (refined, wheat- and oat +wheat-based whole-grain food). The intervention will last 16 weeks, and will comprise three dietary interventions. For the first 4 weeks all volunteers will consume a refined diet to establish a baseline before allocation to the above treatment groups. The choice of 12 weeks for each dietary intake corresponds to the time necessary to observe changes in serum lipid levels and markers of dietary lignans. 180 subjects will be needed and the randomisation will be stratified by gender, age, smoking status and BMI. Compliance will be determined with the analysis of plasma biomarker (enterolactone), and with the analysis of a weekly checklist of whole-grain food consumed. A drop-out of 20% is anticipated and recruitment numbers will take this in to consideration.  **Subjects:**  Men and women aged 40-65 y with a body mass index (in kg/m^2^) between 25 and 35 will be recruited from the surrounding community of Aberdeen. Only subjects sedentary or moderately active (less than two aerobic session per week) will be included. Individuals will be also included if they present signs of metabolic syndrome e.g. if he/she has three or more of the following conditions: fasting plasma glucose (>6.1mm/L), TAG level (>1.7mmol/L), low HDL cholesterol (<1.04 mmol/L for men, < 1.29 mmol/L for women), Hypertension (>130/85 mmHg), central obesity (waist circumference>102 cm for men, > 88cm for women), or if they have moderate hypercholesterolemia.  Individuals will be excluded if they reported to have CVD, diabetes or fasting blood glucose concentration > 7.0mmol/L, asthma, systolic blood pressure >160mm or diastolic blood pressure 99 mm Hg. People with thyroid gland disorders or eating disorders, as well as people taking regularly medication or supplements known to affect any dependant variable measured will be also excluded. Volunteers with high habitual intake of whole-grain foods (> 5 servings per week), as well as people taking regularly nutritional supplements such as antioxidants or fish oil, will be excluded.  **Statistics:**  Expert statistical advice has been sought for the design and future statistical treatment of the data from Graham Horgan, BIOS (Biomathematics and Statistics Scotland), based at the Rowett Research Institute. The experiment is essentially a three-group intervention study, with treatment information assessed between individuals. It will be analysed by using single factor analysis of variance, with baseline measurements and other subject characteristics (age, BMI) as covariates.  Power calculations indicated that for many of the variables such as total cholesterol, LDL cholesterol, ICAM1, apolipoproteins, glucose) we are mainly interested in, variation between individuals has been found by other authors (Jenkins, 2003; Nawawi, 2003: Maki, 2003; Witte, 2003) to be about 10%-20%. Baseline covariate adjustment should reduce this to 5%-10%. This means that 60 subjects per group should give sufficient experimental power (90%) to detect intervention effects of 5%-7%. Even lower variation is expected in total cholesterol (SD approx 0.25mM, range 5-6mM), so that 60 subjects per group will give sufficient power to detect differences of 0.2mM.  **Ethical Approval:**  An application has been submitted to the Grampian Research Ethics Committee and will be forwarded in due course.  **Duration:**  The 180 volunteers will be processed within 40 months.  **Intervention**:  The dietary interventions proposed for this project are designed to compare a diet based on refined cereal products with a) the substitution of 3 servings of refined cereals foods with 3 servings of whole wheat foods and b) the substitution of 3 servings of refined cereals foods with one servings of whole wheat foods and two of oats. The interventions are designed to be practical and realistic for individuals to achieve.  These interventions represent a significant increase in current average intakes of Non Starch Polysaccharides (NSP). It is estimated that the substitution with 3 servings of whole wheat foods will provide approximately 6.4g of NSP per day, of which 2.6 will be soluble fibre, while the whole wheat plus oats intervention will provide approximately 5.3g NSP per day of which 3.6 will be soluble fibre. Data from the 2001/2 Expenditure and Food Survey (http://statistics.defra.gov.uk/esg/publications/efs/default.asp) indicate that the average intake of NSP in Scotland is 12.6g per day. The proposed interventions are likely to bring intakes close to the Dietary Reference Value of 18g per day but will not cause intakes to exceed the upper limit of 24g per day set for individuals (Department of Health, 1991).  Procedures for the dietary intervention: Dietary Patterns Before the start of the study volunteers will be asked to complete the SCG Food Frequency Questionnaire in order to obtain information about their usual dietary patterns. This information will be used to:   1. Exclude volunteers who consume higher than average intakes of whole grain foods (defined as > 5 servings of whole grain foods per week) 2. To assist in explaining the dietary instructions given to each volunteer.  Food Intake Food intake will be measured using a 7 day estimated food record at baseline and during weeks 4 and 9. This will allow us to compare intakes between baseline and intervention and between the two intervention groups. Week 0Food Diary Volunteers will be asked to complete a 7 day estimated food record prior to the start of the intervention (week 0). Weeks 1-4 **Basal Refined Diet (BRD)**  The purpose of the BRD is to standardise the dietary intake of cereals foods across the study population before substituting specific whole grain foods. As we will exclude individuals whose usual intake of whole grain foods exceeds 5 servings a week, the BRD will represent, at most, a decrease in NSP of around 1.5g per day and for many volunteers this will be considerably less. Thus the BRD gives us the advantage of standardising cereal foods across our study volunteers without dramatically altering usual intake of NSP. Food Diary Volunteers will be asked to complete a 7 day estimated food record during week **4** of the study period.  **Weeks 4-16 (Group 1)** Basal Refined Diet Continued Volunteers in Group 1 will be asked to continue as above on the BRD for the duration of the study. Food Diary Volunteers will be asked to complete a 7 day estimated food record during week **9** of the intervention.  **Weeks 4-16 (Group 2)**  **Whole Wheat Diet (WWD)- BRD plus 3 servings of whole wheat foods per day**  Volunteers in Group 2 will be asked to replace 3 servings of refined cereal foods per day with 2 slices of whole wheat bread (70-80g per day) and 1 serving of a prescribed whole wheat breakfast cereal. Volunteers will be provided with a list of suitable 100% wholemeal bread brands available in the supermarkets/retail outlets. In addition, volunteers will be encouraged to check the suitability of any product they are in doubt about with the study staff.  Volunteers will be supplied with *Weetabix* and *Bran Flakes*, which are both high in whole wheat, and asked to eat a serving (30-40g) of either one on a daily basis. Food Diary Volunteers will be asked to complete a 7 day estimated food record during week **9** of the intervention.  **Weeks 4-16 (Group 3)**  Whole Wheat + Oats Diet (WWOD)- BRD plus 1 serving whole wheat and 2 servings of oats per day  Volunteers in Group 3 will be asked to replace 3 servings of refine cereal foods per day with 1 slice of whole wheat bread (35-40g per day) and, instead of the whole wheat breakfast cereals, subjects will be provided with a 60-80g serving of whole grain rolled oats. Half will be provided as one serving of oatmeal, prepared either as porridge or, for example, soaked overnight in milk and served with ingredients of choice (Swiss style, without nuts). Recipe ideas will be supplied. The other half will be provided as oat cakes available from Waitrose, which contains 75% carbohydrate from oats and 9% olive oil as only fat. Food Diary Volunteers will be asked to complete a 7 day estimated food record during week **9** of the intervention.  **Summary of Dietary Interventions**   | Volunteer Group | Dietary Intervention | | --- | --- | | 1 | - Basal Refined Diet for 16 weeks | | 2 | - Basal Refined Diet for 4 weeks - 3 servings of whole wheat foods per day for the following 12 weeks | | 3 | - Basal Refined Diet for 4 weeks - 1 serving of whole wheat foods plus 2 servings of oats per day for the following 12 weeks |   **Volunteers**  Volunteers will be men or female, aged 40 to 65, 25< BMI<35, free from known disease and will not be taking medication or nutritional supplements such as antioxidants or fish oil. During each visit, the subjects will be asked to fill a health questionnaire as well as a questionnaire to determine their physical activity level. Their BMI, age, and blood pressure will be recorded.  Four fasted blood samples of 50 mL will be taken, 1 baseline, 1 at the end of the run-in period and two during the intervention (middle and end). Plasma and serum will be isolated from the blood. Total cholesterol, HDL and LDL cholesterol concentrations, TAG and non esterified fatty acid concentrations, as well as insulin and glucose concentrations will be determined. In addition, the plasma concentrations of apoA1, apo B_100_, homocysteine, C reactive protein, sICAM-1, Il-6, fibrinogen, von Willebrand factor, ghrelin, arginine and methylated metabolites) and enterolactone will be measured. Plasma lipoprotein subclasses particles size and number will be measured by proton nuclear magnetic resonance spectroscopy. In vivo dynamic measurement of vascular tonicity (as measured by arterial stiffness) will be carried out four times during the intervention (baseline, and after each of the intervention) using pulse-wave velocity. Compliance will be assessed by dietary monitoring and also by measuring plasma level of enterolactone. Laboratory Analyses All samples of the same subject will be analysed within the same run to reduce the variance of the results. Randomisation The randomisation of the subjects recruited will be carried out by the Health Research Services Unit. The randomisation will be stratified by gender, age (within 5 y intervals), smoking and BMI. Vascular Tonicity In vivo dynamic vascular function will be assessed by pulse contour analysis (Pulse Trace PCA, Micromedical Ltd), which is well correlated to pulse wave velocity measurements, less expensive and simpler to use. These are particularly good at predicting CVD risk in otherwise low risk individuals within the age group to be studied, as described in several papers and reviews (e.g. Safar ME, 2001, Curr opinion in Nephr. Hypert. 10: 257-261).  **Approach 01:** recruitment of volunteers, randomisation and baseline characteristics  Task 01:  Subtask 01: staff recruitment and training. (first three months)  The University of Aberdeen will be responsible for the staff recruitment (senior dietician and technician Grade D)  Subtask 02: volunteer recruitment  Dr F. Thies, Mrs H. Peace will be responsible for the recruitment of the volunteers. Advertisements will be placed in local papers, and displayed in public areas and social clubs in order to recruit subjects. Advertisement in local radio will also be used. Their randomization will be carried out by the Health Services Research Unit of the University of Aberdeen. One person will be required for this task, which should take 33 months to complete.  Task 02: Weight and blood pressure of the volunteers will be measured during each visit. In addition, volunteers will be asked to measure their weight every two weeks during the dietary intervention. The volunteers will be asked to fill out a questionnaire about their health, level of exercise and medication. Blood pressure will be measured with a OMRON705CP sphygmomanometer accredited by the British Heart Foundation with the subject in the seated position, using the right arm and the appropriate size cuff. BP will be measured at least one hour after the subject last meal and at least 30 min after smoking or consumption of cafeinated beverage. Subjects will remain seated for 5 min prior to each measurement. Three consecutive blood pressure readings will be taken at 30s intervals. The first measurement will be discarded and the following two will be averaged to define the BP measurement for each visit day. Such method has been used previously in dietary intervention studies such as the DASH trial (Ann Epidemiol 1995, 5: 108-118). F. Thies will be responsible for this task. 32 months will be required to achieve this task.  **Approach 02:** Dietary intervention, monitoring and assessment  Task 03: The recruited volunteers will be randomised to one of 3 dietary intervention groups. The diet plan, monitoring and assessment of dietary intake of the subjects are described in detail in the research plan. Every aspect of the dietary phase of the study will be coordinated by Mrs H. Peace. 32 months will be required to achieve this task.  **Approach 03:** in vivo vascular function  Task 04: Pulse-wave velocity measurements will be done at the University of Aberdeen. The person who will carry out the measurement will follow a course prior to the start of the trial on pulse wave velocity run in the vascular research clinic, Addenbrookes hospital, Cambridge. J. Brittenden will be responsible for this task. 32 months will be required to achieve this task.  **Approach 04:** Sample collection and processing  50 mL of fasted blood will be taken from the volunteers during each visit (4 samples in total) and processed immediately.  Task 05: Frank Thies and Klaus Wahle will be responsible for collecting and processing the blood samples. This task will take 32 months to be completed. Blood collection will be processed with minimal use of tourniquet by clean venepuncture with green 21G butterfly or Vacutainer system. Seven mL will be transferred to a plain tube for the analysis of apoA1, apoB, glucose, cholesterol (total and HDL) and insulin). The rest of the blood (43 mL) will be collected into EDTA tubes for the remaining measurements. The samples will be processed and frozen within 2 hours following blood collection.  Subtask 01: The serum from 7 mL of blood will be aliquoted and frozen for future analysis of cholesterol, HDL, LDL, apo A1, apoB_100_, insulin and glucose.  Subtask 02: The rest of the blood (43 mL) will be spun immediately after collection to isolate the plasma, aliquoted and frozen for further analysis.  Task 06: Plasma/serum analyses  Subtask 01: serum lipid and apolipoprotein analyses  All the lipid assays will be carried out using a KONE Selective Chemistry Analyser. Total cholesterol will be analysed using Labmedics kit number 981261 whereby cholesterol esters are hydrolysed by an esterase to cholesterol and fatty acids then oxidised to form a coloured quinoneimine dye which is measured at 510nm. HDL-Cholesterol will be determined using Labmedics kit number 981651 in which cholesterol esterase and cholesterol oxidase are modified by PEG, causing selective catalytic reactivity towards high density lipoprotein fractions. LDL-Cholesterol will be determined using the KONE Instruments LDL-Cholesterol kit (Cat. number 981656) provided by Labmedics Limited. This method uses the selective micellary solubility of LDL_Cholesterol by a non-ionic detergent. Triglycerides will be determined using the KONE Instruments triglyceride kit (Cat. number 981264 provided by Labmedics Ltd whereby lipase, glycerol kinase and glycerol phosphate oxidase catalyses the cleavage of triglycerides with formation of quinoneimine dye read at 510nm. Non-Esterified Fatty Acids (NEFA) will be determined using the Wako NEFA-C kit (Cat. number 994-74509) provided by Alpha Labs. The enzyme / coenzyme system, Aycl-coenzyme A synthetase and Aycl-coenzyme A Oxidase activates and oxidises the FFA to 2,3-trans-Enoyl-CoA and hydrogen peroxide which after further reactions forms a colour product which absorbs at 540nm. Apo A1 and apo B_100_ levels will be determined immuno assay. These measurements will be done at the University of Aberdeen (Department of Clinical Biochemistry, Dr WG Simpson) and the Rowett Research Institute. G. Duthie and F. Thies will be responsible for this task. 21 months will be required to achieve this task.  Subtask 02: lipoprotein subclasses  Lipoprotein concentrations and lipoprotein size will be measured by using proton nuclear magnetic resonance (NMR) spectroscopy. The analyses will be carried out in the Department of Chemistry, University of Aberdeen, in the laboratory of Prof Jaspars. The method of Otvos *et al* (*Clin Chem* **1992**, *38/9*, 1632-8) will be applied to determine concentrations and subspecies distributions of plasma lipoprotein. The method will be calibrated using VLDL, LDL, HDL and protein. These can be obtained from plasma of fasting donors using sequential flotation ultracentrifugation followed by dialysis to concentrate the samples. To mimic the behaviour of subspecies of different diameters, the spectra of these four will be shifted to give roughly 20 components which can be used in a linear least squares fit (deconvolution) of the clinical samples. This approach is necessary because of the unavailability of a sufficient number of lipoprotein subspecies standards, and has been shown to work well previously (*Clin Chem* 1992, *38/9*, 1632-8).  The clinical samples will be frozen after acquisition and thawed when necessary. NMR spectra will be acquired at 45^o^C after the addition of 70 μL D_2_O as internal lock solvent to 630 μL plasma. The region between δ 0.67 - 0.87 ppm will be exported (*ca* 200 datapoints) for each sample and deconvoluted using a linear least squares fitting algorithm using the basis sets generated from the shifted NMR spectra for the four standards. This algorithm, using singular value decomposition, can be implemented using a standard spreadsheet package such as Microsoft’s Excel. The concentrations of each of the components can then be determined. Previous studies have shown this methodology compares favourably with the alternative and much more technically challenging ultracentrifugation and nondenaturing gradient gel electrophoresis. F. Thies will be responsible for this task. 21 months will be required to achieve this task.  Subtask 03: Insulin sensitivity  Mathematical combinations of fasting insulin and glucose measures (logHOMA-IR and revised-QUICKI, which takes into account non-esterifies fatty acids concentrations) provide estimates of insulin sensitivity comparable to “Gold standard” hyperinsulinaemic clamp and are superior to measures based on insulin alone (Hunt et al, 2001, J.Endocrin. Metab. 86: 5457-5462, Brady LM et al., 2004, Inter. J. Obesity 28: 222-227). Fasted serum glucose and insulin concentrations will be measured respectively by an automated enzymatic method and microparticle enzyme immunoassay (Abbott Laboratories). Analyses will be carried out in the Department of Clinical Biochemistry (Royal Aberdeen Hospital). F. Thies will be responsible for this task. 21 months will be required to achieve this task.  Subtask 04: homocysteine, folate and arginine metabolites  Plasma folate will be determined using a commercially available kit (Simultra Radioimmunoassay kit [125 l] supplied by Flow, UK. Total homocysteine in plasma will be measured by reverse phase HPLC using a DS30 Homocysteine Assay kit in combination with a DS30 anaylser Drew Scientific, UK) Dr. S. Duthie of the Rowett Institute will be responsible for this task. Arginine and methylated metabolites will be measure by HPLC.  Subtask 05: markers of inflammation  Ultra sensitive C reactive protein will be measured by an ultra sensitive, double antibody ELISA currently used in the Department of Clinical Biochemistry (Royal Aberdeen Hospital). Von Willebrand factor, soluble adhesion molecules (sICAM-1) and IL-6 will be measured by ELISA. Fibrinogen will be measured using the Clauss method (Thrombin clotting method). K. Wahle, F. Thies will be responsible for this task. 21 months will be required to achieve this task  Subtask 06: enterolactone.  Enterolactone will be determined using the Time-Resolved FluoroImmunoAssay (Labmaster 1212-2001) in which goat anti-rabbit IgG binds to anti-enterolactone antibody. Europium-labelled enterolactone and sample enterolactone compete for the antibody and resulting dossociated Europium ions form a fluorescent chelate which is inversely proportional to the concentration of enterolactone in the sample. Dr. G. Duthie will be responsible for this task. 21 months will be required to achieve this task.  **Approach 05:** Complete data preparation/ statistical analysis and begin final report.  Task 07: All centres are responsible to verify and provide their data. Statistical analysis will be carried out by BIOS (Biomathematics and Statistics Scotland), based at the Rowett Research Institute. Collation of data will be the responsibility of F. Thies after discussion with the management committee. Dissemination of data and publication in peer-reviewed scientific journals will be mutually agreed within the management committee. 6 months will be required to achieve this task. |
